# Association between uric acid and cardiac outcomes mediated by neutrophil-to-lymphocyte ratio in patients with left ventricular diastolic dysfunction and pulmonary hypertension

**DOI:** 10.1038/s41598-024-53077-1

**Published:** 2024-02-02

**Authors:** Ping Du, Xin Gao, Qiaobing Sun, Minghui Gong, Yu Pan, Qinpeng Guo, Xu Zhao, Ran Guo, Yan Liu

**Affiliations:** 1 Department of Cardiology, The First Affiliated Hospital of Dalian Medical University, Dalian, China; 2 Department of Cardiovascular Ultrasound, The First Affiliated Hospital of Dalian Medical University, Dalian, China

**Keywords:** Cardiology, Interventional cardiology

## Abstract

To evaluate the association of uric acid (UA) with adverse outcomes and its potential mediator in patients with left ventricular diastolic dysfunction (LVDD) and pulmonary hypertension (PH). We retrospectively analyzed 234 patients with LVDD and PH. The baseline characteristics of patients with low UA (≤ 330 µmol/L) group were compared with high UA (> 330 µmol/L) group. Adverse outcomes included all-cause mortality, cardiac death and heart failure (HF) hospitalization. Their association with UA and the mediator were evaluated using Cox regression and mediation analysis. The mediation proportion was further quantified by the R mediation package. During a mean follow-up of 50 ± 18 months, there were 27 all-cause deaths, 18 cardiovascular deaths and 41 incidents of HF hospitalization. Multivariable Cox regression analysis showed UA was an independent risk factor of adverse outcomes in LVDD and PH patients, even after adjusting for age, sex, body mass index, medical histories, systolic blood pressure, fasting blood glucose, total cholesterol, triglyceride, eGFR, BNP and medications. The hazard ratios (HRs) for UA (per 10 µmol/L increase) were as below: for all-cause mortality, HR 1.143, 95% CI 1.069–1.221, *P* < 0.001; for cardiac death, HR 1.168, 95% CI 1.064–1.282, *P* = 0.001; for HF hospitalization, HR 1.093, 95% CI 1.035–1.155, *P* = 0.001. Neutrophil-to-lymphocyte ratio (NLR) played a partial mediation role in the association, and the mediation proportion for NLR on the UA-adverse outcomes were 21%, 19% and 17%, respectively. In patients of LVDD with PH, higher UA level was independently correlated with adverse outcomes. Furthermore, NLR partially mediated the effect of UA on the risk of all-cause mortality, cardiac death and HF hospitalization.

## Introduction

Left heart disease-related pulmonary hypertension (PH) is the most common type, comprising 65–80% of all PH cases. In heart failure (HF) with reduced ejection fraction (HFrEF), the estimated prevalence of PH is 40–72%, while in heart failure with preserved ejection fraction (HFpEF), the estimated prevalence of PH is 36–83%^1^. Left ventricular (LV) diastolic dysfunction (LVDD) plays an important role in the development of PH, and the occurrence of PH is considered as a predictive marker of poor prognosis.

Hyperuricemia is a common occurrence in cardiovascular diseases such as HF and PH. Several previous studies have revealed that serum uric acid (UA), known as the degrading end product of purine, is associated with cardiovascular diseases and served as a significant risk factor for poor prognosis in hypertension, HF, coronary heart disease (CHD), atrial fibrillation (AF) and PH^2–7^. UA has been known to induce cellular inflammation and oxidative stress^8,9^. UA-induced cardiovascular damage has also been reported to be mediated by several mechanisms, including inflammation, oxidative stress, and activation of the renin–angiotensin–aldosterone and sympathetic nervous systems^10–12^. Additionally, inflammatory mechanisms are involved in the development of LV remodeling, and the systemic inflammatory state is correlated with cardiovascular diseases, including LVDD and PH. Elevated UA triggers a systemic inflammatory state, potentially leading to HFpEF^13^. In the serum UA production process, the generation of oxygen free radicals initiates an inflammatory reaction^14^. Hyperuricaemia contributes to the progression of cardiovascular pathologies by promoting tissue deposits and intracellular uric acid accumulation, ultimately fostering chronic inflammation^10^*.* Moreover, the neutrophil-to-lymphocyte ratio (NLR), a readily detectable inflammatory marker, has been reported to be associated with LV hypertrophy (LVH), and has implications for risk stratification and prognosis in HFpEF and PH^15–17^. Hence, additional investigation is warranted to explore the correlation between UA, NLR and cardiovascular diseases.

However, the relationship between UA and LVDD as well as PH has not been extensively investigated. Therefore, the objective of this study was to evaluate the association of UA with adverse outcomes and its potential mediator in patients with LVDD and PHT. Furthermore, we aimed to elucidate the role of NLR in the correlation between UA and these cardiovascular conditions.

## Methods

### Study population and data collection

**Figure 1:**
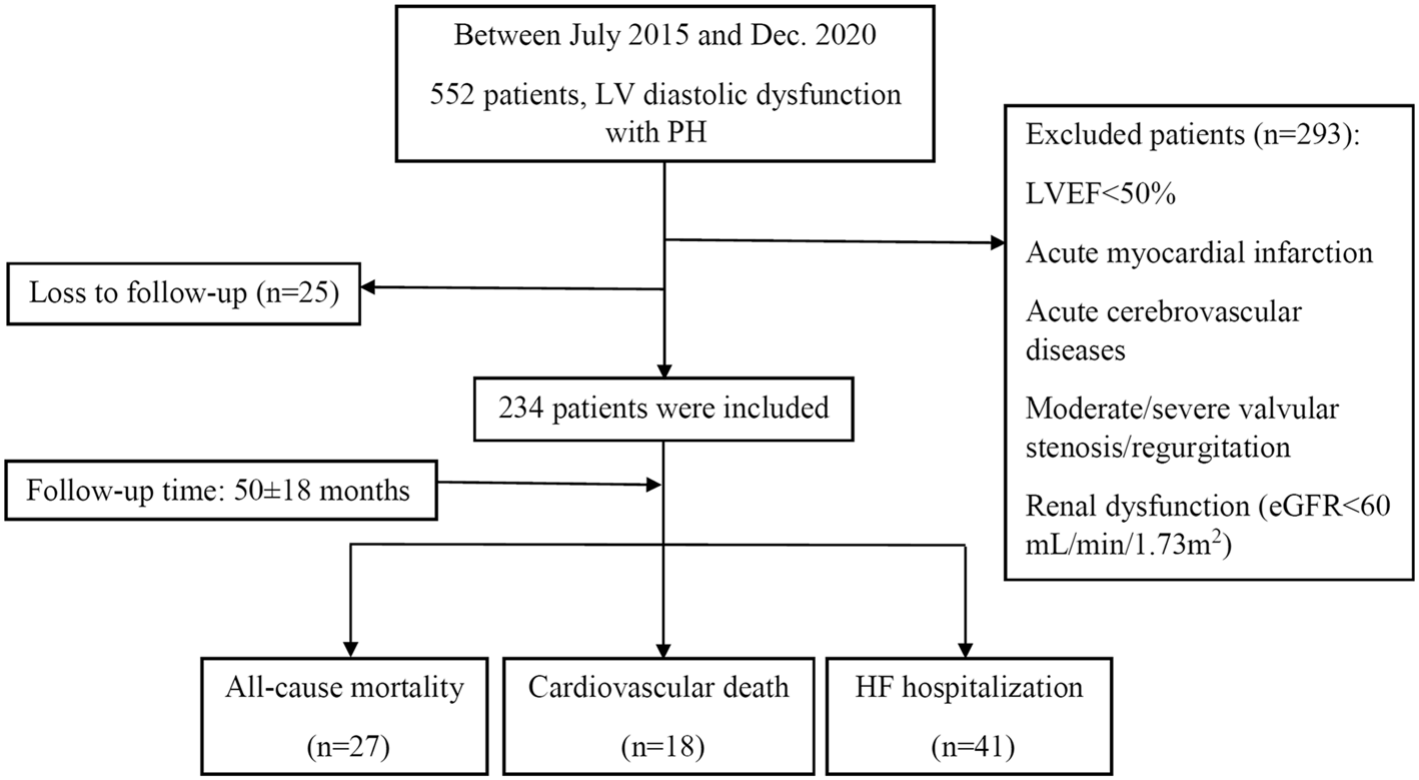
Flowchart of the study population. *LV* left ventricular, *PH* pulmonary hypertension, *LVEF* left ventricular ejection fraction, *eGFR* estimated glomerular filtration rate, *HF* heart failure.

This study involved a retrospective analysis of medical records from the Cardiac Department of the First Affiliated Hospital of Dalian Medical University. A total of 234 patients diagnosed with LVDD and PH between July 2015 and December 2020 were included. The selection criteria required patients to have undergone echocardiography examination during hospitalization and received an echocardiographic diagnosis of LVDD and PH. The exclusion criteria encompassed the following: active infections, patients with LVEF less than 50%, acute myocardial infarction, acute cerebrovascular diseases, moderate or severe cardiac valvular stenosis or regurgitation, renal dysfunction, indicated by the estimated glomerular filtration rate (eGFR) below 60 mL/min/1.73m^2^, and malignant tumors (Fig. 1). Ethical considerations were adhered to, with the study complying with the Declaration of Helsinki, and obtaining approval from the Ethics Committee of the First Affiliated Hospital of Dalian Medical University. Informed consent was obtained from all patients in this study.

### Study endpoints

The study endpoints encompassed three outcomes: all-cause mortality, cardiac death and HF hospitalization. Follow-up commenced upon discharge from the hospital, and data were retrospectively gathered from patients’ medical records, clinic follow-up visits, or telephone interviews.

### Biochemical tests

Venous blood samples were collected from all patients in the morning, following a fasting period of over 8 h. The blood samples were sent to the Laboratory Center at the First Affiliated Hospital of Dalian Medical University for analysis. The parameters measured included blood routine items, serum creatinine (Scr), blood lipids, uric acid (UA), B-type natriuretic peptide (BNP) and high-sensitivity cardiac troponin I (hs-cTnI). Furthermore, the NLR and platelet-lymphocyte-ratio (PLR) were derived from the obtained blood routine values. Renal function was determined via the eGFR, which was determined using the following formula: eGFR (mL/min/1.73 m^2^) = 186 × (Scr/88.402)^−1.154^ × age^−0.203^ (× 0.742 for females).

### Transthoracic echocardiographic evaluation

Echocardiography was conducted using a Vivid E9 ultrasound system (GE Vingmed Ultrasound, Horten, Norway), adhering to the guideline set forth by the American Society of Echocardiography for accurate and standardized measurements^18^. On the LV parasternal long axis view, several measurements were taken, including the right ventricular (RV) diastolic diameter (RVDd), LV diastolic diameter (LVDd), interventricular septum thickness (IVST), LV posterior wall thickness (LVPWT), and left atrial diameter (LAD). To calculate the LV mass (LVM), the following formula was applied: LVM = 0.8 × 1.04 [(LVDd + IVST + LVPWT)^3^ − LVDd^3^] + 0.6. Additionally, the LVMI (g/m^2^) was obtained by dividing LVM by the body surface area (BSA). The main pulmonary artery diameter (PAD) was assessed on the parasternal short axis view. Pulsed-wave flow Doppler was used to measure the early diastolic transmitral flow velocity (E). Additionally, pulsed-wave tissue Doppler was utilized to obtain the lateral mitral myocardial early diastolic velocity (e’) at the apical four-chamber view. The E/e’ ratio was then calculated based on these measurements. The diagnosis of LVDD followed the guideline provided by the American Society of Echocardiography and the European Association of Cardiovascular Imaging. In short, the diagnosis was established if two out of four conditions were met: average E/e’ > 14, septal e’ velocity < 7 cm/s or lateral e’ velocity < 10 cm/s, TR velocity > 2.8 m/s and LA enlargement. To diagnose PH, the tricuspid regurgitation (TR) velocity was evaluated. A cutoff value of 2.8 m/s, corresponding to a systolic pulmonary artery pressure (sPAP) of 36 mmHg was defined as PH^1^ .

### Statistical analysis

Data normality was evaluated through the Kolmogorov–Smirnov test. Continuous variables were reported as either mean ± standard deviation (SD) or the median and interquartile range (IQR; 25th-75th percentile), depending on the distribution of each variable. Differences between two independent groups of continuous variables that followed a normal distribution were assessed using independent-samples T test. For non-normally distributed continuous variables, the Mann–Whitney U test was employed. Categorical variables were analyzed using the Chi-Square test. Statistical significance was defined as a p-value less than 0.05. Kaplan–Meier curves were constructed to illustrate the association between higher and lower UA groups, categorized based on the median of UA, and the corresponding outcomes. Univariable and multivariable Cox regression were conducted to assess the impact of UA on the outcomes. Two models were developed for this purpose. Model 1 was adjusted for age, sex, and body mass index (BMI). In Model 2, additional variables included the history of hypertension, diabetes mellitus (DM), CHD and AF, as well as BNP, fasting plasma glucose (FPG), total cholesterol (TC), triglyceride (TG), eGFR and the use of angiotensin-converting enzyme inhibitor (ACEI)/ angiotensin receptor blocker (ARB)/ angiotensin receptor-neprilysin inhibitor (ARNI), β-blocker, diuretics and antisterone. In addition, the assessment of potential linear or nonlinear associations between UA and clinical outcomes utilized restricted cubic splines with 4 knots, as demonstrated by the “rms” package. Mediation analysis was conducted to investigate the role of NLR in the impact of UA on outcomes. The presence of a mediation effect was considered if the following three conditions were met: 1. UA showed a significant association with NLR in the multiple linear regression model. 2. UA was an independent predictor for outcomes in the multivariable Cox regression excluding NLR. 3. NLR independently predicted the outcomes in the multivariable Cox regression model, which also included UA. Furthermore, the R mediation package was utilized to estimate the proportion of mediation effect of UA on outcomes, which was mediated through NLR. Statistical analysis was performed using SPSS software 21.0 (SPSS, Chicago, Illinois, USA) and R (version 4.2.2, R Foundation for Statistical Computing, Vienna, Austria).

## Results

### Baseline characteristics

A total of 234 patients were stratified into two groups using the median of serum UA level: Group I (n = 117) comprised patients with UA ≤ 330 µmol/L, and Group II (n = 117) comprised patients with UA > 330 µmol/L. Table 1 presents a summary of the baseline characteristics, biochemical tests and echocardiographic parameters of the patients. Both groups exhibited similar age, gender, BMI, smoking and drinking habits, medical histories of DM, CHD, and AF, as well as heart rate (HR). However, Group I had higher systolic blood pressure (SBP) and diastolic blood pressure (DBP) compared to Group II (*P* < 0.05). In terms of routine blood biochemistry tests, Group II showed significantly increased levels of TC, TG, low-density lipoprotein cholesterol (LDL-C) and Scr compared to Group I (*P* < 0.05). Regarding cardiovascular medications, both groups had similar usage rates of ACEI/ARB/ARNI, β-blockers, CCB, diuretics, antisterone, aspirin, statins and SGLT2 inhibitors.

**Table 1:** Clinical characteristics of patients.

	All (n = 234)	Group I (UA ≤ 330umol/L, n = 117)	Group II (UA > 330umol/L, n = 117)	*P* value
Age (years)	69 ± 7	70 ± 7	69 ± 8	0.470
Male sex, n (%)	71 (30%)	29 (24.8%)	42 (35.9%)	0.088
BMI (kg/m^2^)	25.02 ± 3.14	24.82 ± 3.29	25.23 ± 2.99	0.313
Smokers, n (%)	29 (12%)	13 (11.1%)	16 (13.7%)	0.692
Drinkers, n (%)	25 (11%)	9 (7.7%)	16 (13.7%)	0.203
Medical history				
Hypertension, n (%)	194 (83%)	106 (90.6%)	88 (75.2%)	**0.003**
DM, n (%)	80 (34%)	42 (35.9%)	38 (32.5%)	0.679
CHD, n (%)	171 (73%)	87 (74.4%)	84 (71.8%)	0.768
AF, n (%)	93 (40%)	43 (36.8%)	50 (42.7%)	0.423
Clinical characteristics				
SBP (mmHg)	151 ± 24	154.52 ± 25.51	147.60 ± 21.00	**0.024**
DBP (mmHg)	80 ± 13	81.29 ± 13.16	77.87 ± 12.62	**0.044**
HR (bpm)	70 (60,78)	70 (62, 80)	70 (60, 76)	0.478
FPG (mmol/L)	5.40 (4.80, 7.01)	5.29 (4.77, 6.90)	5.45 (4.83, 6.99)	0.854
TC (mmol/L)	4.65 ± 1.10	4.51 ± 1.08	4.80 ± 1.11	**0.047**
TG (mmol/L)	1.29 (0.93, 1.77)	1.23 (0.91, 1.64)	1.38 (0.98, 1.91)	**0.019**
HDL-C (mmol/L)	1.16 ± 0.28	1.19 ± 0.30	1.12 ± 0.25	0.068
LDL-C (mmol/L)	2.60 ± 0.79	2.47 ± 0.75	2.72 ± 0.80	**0.016**
Scr (μmol/L)	66.07 ± 14.06	63.36 ± 12.88	68.78 ± 14.72	**0.003**
eGFR (ml/min/1.73 m^2^)	93.86 ± 19.90	96.34 ± 20.44	91.37 ± 19.12	0.056
BNP (ng/L)	91.26 (49.44, 190.42)	92.93 (52.02.44, 244.73)	87.99 (48.29, 180.21)	0.480
Hs-cTnI (μg/L)	0.02 (0.01, 0.05)	0.02 (0.01, 0.05)	0.02 (0.01, 0.05)	0.933
NLR	2.12 (1.53, 3.11)	2.12 (1.40, 3.04)	2.12 (1.71, 3.55)	0.216
PLR	112.05 (84.16, 148.04)	115.57 (86.97, 150.24)	108.88 (82.01, 147.18)	0.531
Medications				
ACEI/ARB/ARNI (%)	145 (62%)	77 (65.8%)	68 (58.1%)	0.281
β-blockers (%)	142 (61%)	65 (55.6%)	77 (65.8%)	0.070
CCB (%)	134 (57%)	73 (62.4%)	61 (52.1%)	0.146
Diuretics (%)	86 (37%)	37 (31.6%)	49 (41.9%)	0.136
Antisterone (%)	70 (30%)	29 (24.8%)	41 (35.0%)	0.116
Aspirin (%)	134 (57%)	70 (59.8%)	64 (54.7%)	0.509
Statins (%)	177 (76%)	91 (77.8%)	86 (73.5%)	0.543
SGLT2i (%)	10 (4.3%)	7 (6.0%)	3 (2.6%)	0.333
Echocardiographic data				
RVD (mm)	18 (16, 20)	17 (16, 19)	18 (17, 20)	**0.001**
LAD (mm)	41 (38, 44)	40 (38, 43)	42 (38, 45)	0.095
LVMI (g/m^2^)	76 ± 17	75.41 ± 13.43	77.40 ± 19.13	0.357
LVEF (%)	58 (57, 59)	58 (57.5, 59)	58 (56, 59)	0.181
E/e’ ratio	14 (13, 16)	14.0 (12.3, 14.45)	15.0 (13.2, 17.0)	** < 0.001**
PAD (mm)	23 (22, 25)	23 (22, 25)	23 (22, 25)	0.808
RVSP (mm)	43 (39, 48)	42 (39, 47)	44 (39, 51)	0.133
Adverse outcomes				
All-cause mortality, n (%)	27 (11.5%)	4 (3.4%)	23 (19.7%)	** < 0.001**
Cardiac death, n (%)	18 (7.7%)	2 (1.7%)	16 (13.7%)	**0.001**
HF hospitalization, n (%)	41 (17.5%)	9 (7.7%)	32 (27.4%)	** < 0.001**

Significant values are in bold.

*UA* uric acid, *BMI* body mass index, *DM* diabetes mellitus, *CHD* coronary heart disease, *AF* atrial fibrillation, *SBP* systolic blood pressure, *DBP* diastolic blood pressure, HR heart rate, *FPG* fasting plasma glucose, *TC* total cholesterol, *TG* total triglyceride, *HDL-C* high-density lipoprotein cholesterol, *LDL-C* low-density lipoprotein cholesterol, *Scr* serum creatinine, *eGFR* estimated glomerular filtration rate, *BNP* B-type natriuretic peptide, *Hs-cTnI* high-sensitivity cardiac troponin I, *NLR* neutrophil-to-lymphocyte ratio, *PLR* platelet-to-lymphocyte ratio, *ACEI* angiotensin- converting enzyme inhibitor, *ARB* angiotensin receptor blocker, *ARNI* angiotensin receptor neprilysin inhibitor, *CCB* calcium channel blocker, *SGLT2i* sodium-glucose cotransporter 2 inhibitor, *RVD* right ventricular diameter, *LAD* left atrial diameter, *LVMI* left ventricular mass index, *LVEF* left ventricular ejection fraction, *E/e’* ratio of early diastolic transmitral velocity to early diastolic mitral annular velocity, *PAD* pulmonary artery diameter, *RVSP* right ventricular systolic pressure, *HF* heart failure.

In terms of echocardiographic data, there were no significant differences observed between the two groups in LAD, LVMI, LVEF, PAD, and right ventricular systolic pressure (RVSP). However, Group II had significantly higher RVD and E/e’ ratio compared to Group I (*P* ≤ 0.001).

### The predictive values of UA for outcomes

**Figure 2:**
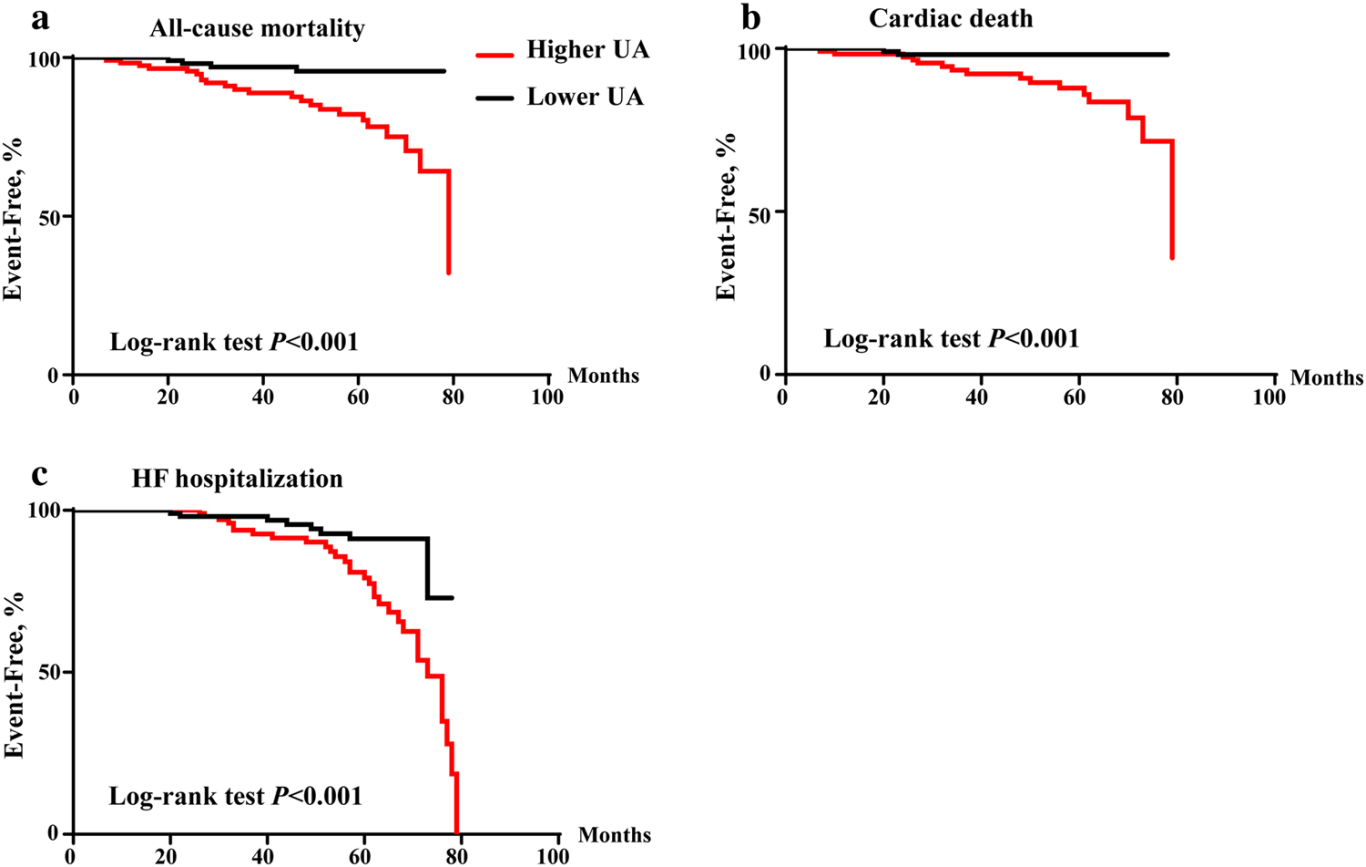
Kaplan–Meier curve of all-cause mortality (**A**), cardiac death (**B**) and HF hospitalization (**C**). *HF* heart failure, *UA* uric acid.

The average follow-up was 50 ± 18 months, and the Kaplan–Meier curve of outcomes are shown in Fig. 2. Higher UA levels exhibited a significantly associated with all outcomes: Group II demonstrated a higher all-cause mortality rate of 19.7% compared to 3.4% in Group I (*P* < 0.001); cardiac death occurred in 13.7% of Group II, whereas it was only 1.7% in Group I (*P* = 0.001); HF hospitalization rates were 27.4% in Group II compared to 7.7% in Group I (*P* < 0.001) (Table 1). We employed univariate and multivariable cox regression analyses to assess the impact of UA on outcomes of patients with LVDD and PHT. The findings revealed that higher UA level was identified as an independent predictor of various outcomes, including all-cause mortality, cardiac death and HF hospitalization. These associations remained statistically significant even after adjusting for multiple clinical factors, such as age, gender, BMI, medical histories, SBP, BNP, FPG, TC, TG, and the use of ACEI/ARB/ARNI, β-blocker, diuretics and antisterone. The hazard ratios (HR) for the aforementioned outcomes in relation to UA (per 10 µmol/L) were as follows: 1.143 (95% CI 1.069–1.221) with a statistical significance (*P* < 0.001), 1.168 (95% CI 1.064–1.282) with a statistical significance (*P* = 0.001), and 1.093 (95% CI 1.035–1.155) with a statistical significance (*P* = 0.001) respectively (Table 2). According to the results, an increase of 10 µmol/L in UA concentration corresponded to a 14.3% increase in all-cause mortality, a 16.8% increase in cardiac death, and a 9.3% increase in HF hospitalization.

**Table 2:** Predictive values of UA (per 10 µmol/L) for adverse outcomes.

	Univariable Cox regression model		Multivariable Cox regression		Multivariable Cox regression	
			Model 1		Model 2	
	HR (95% CI)	*P* value	HR (95% CI)	*P* value	HR (95% CI)	*P* value
All-cause mortality	1.077 (1.045–1.109)	< 0.001	1.106 (1.060–1.153)	< 0.001	1.143 (1.069–1.221)	< 0.001
Cardiac death	1.081 (1.043–1.120)	< 0.001	1.090 (1.042–1.140)	< 0.001	1.168 (1.064–1.282)	0.001
HF hospitalization	1.054 (1.024–1.084)	< 0.001	1.062 (1.025–1.099)	0.001	1.093 (1.035–1.155)	0.001

Model 1, adjustment for age, sex and BMI.

Model 2, adjustment for Model 1 plus hypertension, DM, CHD, AF, SBP, BNP, FPG, TC, TG, eGFR, and the use of ACEI/ARB/ARNI, β-blocker, diuretics and antisterone.

*UA* uric acid, *HF* heart failure, *HR* hazard ratio, *BMI* body mass index, *DM* diabetes mellitus, *CHD* coronary heart disease, *AF* atrial fibrillation, *SBP* systolic blood pressure, *BNP* B-type natriuretic peptide, *FPG* fasting plasma glucose, *TC* total cholesterol, *TG* total triglyceride, *eGFR* estimated glomerular filtration rate, *ACEI* angiotensin- converting enzyme inhibitor, *ARB* angiotensin receptor blocker, *ARNI* angiotensin receptor neprilysin inhibitor.

### The nonlinearity of UA and clinical outcomes

In this study, we also investigated the nonlinear relationship of UA with clinical outcomes. After adjusting for clinical confounding factors in Model 2, there were observed approximate linear relationships (*P*_*nonlinear*_ > 0.05 for all outcomes).

### The predictive values of NLR for outcomes

We also performed univariate and multivariable cox regression analyses to evaluate the impact of NLR on outcomes of patients with LVDD and PHT. Our findings indicated that higher NLR level was an independent predictor of various outcomes, including all-cause mortality, cardiac death and HF hospitalization. These associations remained statistically significant even after adjusting for multiple clinical factors, such as age, gender, BMI, medical histories, SBP, BNP, FPG, TC, TG, UA, and the use of ACEI/ARB/ARNI, β-blocker, diuretics and antisterone. The hazard ratios (HR) for the aforementioned outcomes in relation to NLR were as follows: 1.238 (95% CI 1.096–1.398) with a statistical significance (*P* = 0.001), 1.286 (95% CI 1.105–1.497) with a statistical significance (*P* = 0.001), and 1.163 (95% CI 1.055–1.282) with a statistical significance (*P* = 0.002) respectively (Table 3).

**Table 3:** Predictive values of NLR for adverse outcomes.

	Univariable Cox regression model		Multivariable Cox regression		Multivariable Cox regression	
			Model 1		Model 2	
	HR (95% CI)	*P* value	HR (95% CI)	*P* value	HR (95% CI)	*P* value
All-cause mortality	1.164 (1.095–1.237)	< 0.001	1.172 (1.088–1.262)	< 0.001	1.238 (1.096–1.398)	0.001
Cardiac death	1.171 (1.090–1.258)	< 0.001	1.174 (1.075–1.281)	< 0.001	1.286 (1.105–1.497)	0.001
HF hospitalization	1.111 (1.047–1.179)	0.001	1.122 (1.048–1.201)	0.001	1.163 (1.055–1.282)	0.002

Model 1, adjustment for age, sex and BMI.

Model 2, adjustment for Model 1 plus hypertension, DM, CHD, AF, SBP, UA, BNP, FPG, TC, TG, eGFR, and the use of ACEI/ARB/ARNI, β-blocker, diuretics and antisterone.

*NLR* neutrophil-to-lymphocyte ratio, *HF* heart failure, *HR* hazard ratio, *BMI* body mass index, *DM* diabetes mellitus, *CHD* coronary heart disease, *AF* atrial fibrillation, *SBP* systolic blood pressure, *UA* uric acid, *BNP* B-type natriuretic peptide, *FPG* fasting plasma glucose, *TC* total cholesterol, *TG* total triglyceride, *eGFR* estimated glomerular filtration rate, *ACEI* angiotensin- converting enzyme inhibitor, *ARB* angiotensin receptor blocker, *ARNI* angiotensin receptor neprilysin inhibitor.

### Mediation effect analysis

**Figure 3:**
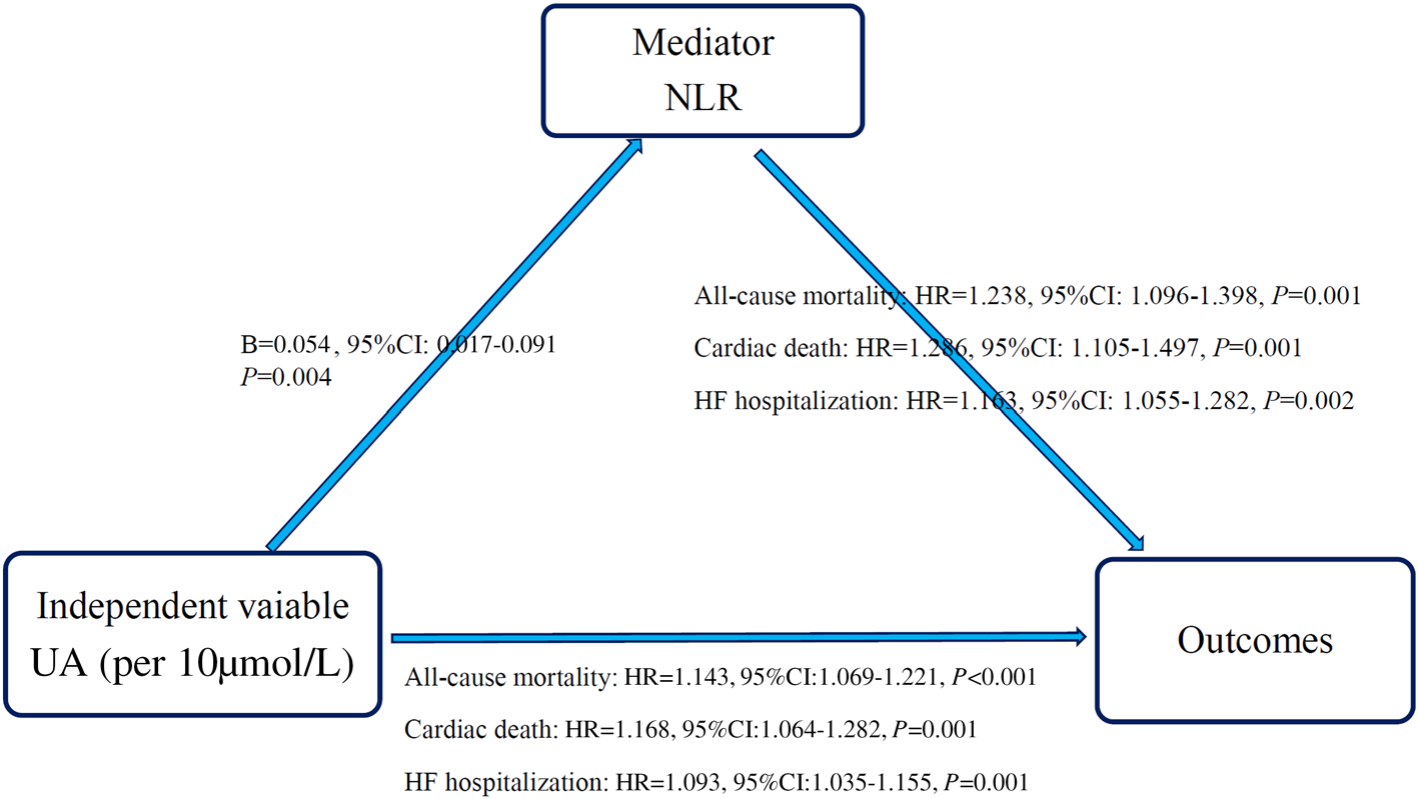
Mediating effect by the NLR between UA and outcomes. *NLR* neutrophil-to-lymphocyte ratio, *UA* uric acid, *HF* heart failure.

Figure 3 presents the findings from our causal mediation analysis. Firstly, we observed a significant linear relationship between UA (independent variable) and NLR (mediator) through multiple linear regression analysis. Secondly, when NLR was not included in the multivariable Cox regression model, UA was an independent predictor for the outcome variables. Thirdly, both UA and NLR continued to exhibit significance in predicting the outcome variables when included together in the multivariable Cox regression model. Thus, this comprehensive mediation analysis confirmed the partial mediation role of NLR in the association between elevated UA levels and increased risks of all-cause mortality, cardiac death and HF hospitalization.

To assess the mediation proportion of NLR on the relationship between UA and various outcomes, we further utilized the R mediation package. The mediation proportion of NLR for the association between UA and all-cause mortality was 21% (95% CI 4.42–51.0%, *P* = 0.014), for the association between UA and cardiac death was 19% (95% CI 1.49–57.0%, *P* = 0.036), and for the association of UA and HF hospitalization was 17% (95% CI 1.08–46.0%, *P* = 0.044).

## Discussion

In this retrospective study conducted at a single center, several main findings were observed. Firstly, higher UA level was identified as an independently risk factor for adverse outcomes, including all-cause mortality, cardiac death and HF hospitalization, in patients with LVDD and PH. Additionally, we found that NLR, a classic and easily-obtainable inflammatory biomarker, partially mediated the association between UA and the occurrence of these adverse outcomes in these patients.

As well known, the clinical classification of PH includes 5 groups: pulmonary artery hypertension (PAH) (Group 1), PH associated with left heart disease (Group 2), PH associated with lung diseases (Group 3), PH associated with pulmonary artery obstructions (Group 4), and PH with unclear/multifactorial mechanisms (Group 5). Group 2 PH, also known as post-capillary PH, is commonly seen in patients with HF (HFrEF and HFpEF) and valvular heart disease. The development of PH associated with left heart disease primarily results from chronic elevation of LA pressure, which is then transmited to the pulmonary circulation, initially causing isolated post-capillary PH (Ipc-PH). However, as the entire pulmonary vasculature undergoes remodeling and pulmonary vascular resistance increases, combined pre- and post-capillary PH (Cpc-PH) develops. In the present study, the PH patients included in our analysis belonged to Group 2 PH. However, they only exhibited LVDD and did not meet the criteria for HFpEF, as some of them did not experience the symptom of dyspnea, and had normal BNP levels. Apart from hemodynamic changes in the left heart itself, non-cardiac factors also contributed to the development of PH.

Metabolic factors play significant roles in the development of PH^19^. Previous studies have reported a high prevalence of metabolic syndrome, up to 39%, in patients with PH, especially in group 2 PH, where the percentage was even higher^20^. Metabolic elements contribute to the occurrence of PH through various mechanisms, including oxidative injury, inflammatory reactions, insulin resistance, endothelial dysfunction and the development of atherosclerotic plaques ^21^. Hyperuricemia, which is commonly associated with metabolic disorders, has been recognized as a risk factor for cardiovascular diseases^22^. Previous studies^23,24^ have shown that hyperuricemia predicts the prognosis in HFpEF patients, and reducing UA levels has a positive impact on these patients’ prognosis. Gu et al.’s study^25^ revealed that hyperuricemia was correlated with the incidence of new-onset of HFpEF and major adverse cardiovascular events (MACE) in patients with hypertension and LVH. In addition, hyperuricemia has also been reported to be independently predict the poor prognosis of PH^7,26,27^. Despite remaining debates, the relationship between UA and LVDD has been explored in various studies. Positive associations have been observed between UA and LVDD in conditions such as hypertension^28^, among healthy young adults^29^ and in women with preserved ejection fraction^30^. Additionally, this association has been noted in patients of heart failure with reduced ejection fraction^31^ or with chronic kidney disease (CKD)^32^. However, in chronic coronary syndromes, there seems to be absence of a significant role for UA in relation to LVDD^33^. These variations in findings may be attributed to the heterogeneity of the study population, the severity of LV remodeling, and the sensitivity of the detection methods employed. In the present study, for the LVDD with PH patients, we found that higher serum UA level (> 330 µmol/L) was an independently predictor of adverse outcomes, including all-cause mortality, cardiac death and HF hospitalization, even after adjusting for age, BMI, hypertension, DM and the other clinical confounders.

Serum UA aggravates cardiovascular damage through various mechanisms, among them, apart from the production of reactive oxygen species (ROS), inflammatory reactions also played a crucial role in it^7,34^. NLR which can be easily obtained from a routine blood test, has been considered as a systemic inflammatory factor. Previous studies have already found higher NLR was associated with adverse outcomes of cardiovascular disease^15,35,36^. In this study, we used R package for mediation analysis, and demonstrated that NLR played a partially mediating role in the relationship between UA and adverse outcomes in patients with LVDD and PH.

Overall, our findings highlighted the importance of metabolic factors, particularly hyperuricemia, in the prognosis of LVDD and PH. Furthermore, NLR served as a potential mediator in the association between UA and adverse outcomes in this patient population.

### Clinical implications

The findings of our study have important clinical implications. We observed a significant association between even mildly elevated UA levels and poor prognosis in patients with LVDD and PH. This highlighted the importance of monitoring UA levels in clinical practice and taking early action to manage elevated UA levels in these patients.

Furthermore, our study suggested that systemic inflammatory reaction may play a mediating role in the association between UA and adverse outcomes. Inflammatory biomarkers, such as NLR, high-sensitivity C-reactive protein (hs-CRP) and interleukin-6 (IL-6), may provide valuable supplementary information to further understand the inflammatory status in these patients. Incorporating these biomarkers into clinical practice could enhance risk stratification and help guide treatment decisions. It is noteworthy that recent studies have brought attention to a novel biomarker, the systemic immune-inflammation index (SII), which integrates information from inflammatory parameters. This index has been recognized to be associated with both cardiac and non-cardiac disorders^37–39^. The SII, theoretically offering a more reliable and comprehensive representation of immune and inflammatory status^40,41^, warrants further investigation in future clinical trials.

Overall, our study emphasizes the need for clinicians to be vigilant about elevated UA levels in LVDD with PH patients and to consider the role of inflammation in their management. By addressing these factors early on, clinicians can provide more targeted and effective treatments, and potentially improve patient outcomes.

## Limitations

There were several limitations for the current study. Firstly, this was a single-center, retrospective study with a limited number of patients. The findings from this study would benefit from confirmation through large-scale clinical trials involving multiple centers. Secondly, some patients in the group 2 PH category had normal resting sPAP as measured by right heart catheterization (RHC), but showed elevated sPAP during exercise. In our center, patients with LVDD, even with PH, did not routinely undergo RHC. Therefore, the diagnosis of PH in these patients relied solely on noninvasive evaluation of RVSP using echocardiography. This may have introduced some measurement variability and limited the accuracy of PH diagnosis. Thirdly, we only assessed the systemic inflammatory state using routine blood tests, and did not test the other specific inflammatory biomarkers, such as hs-CRP, IL-1, IL-6 and others. This may have limited our ability to comprehensively evaluate the inflammatory status of the patients.

## Conclusions

In patients with LVDD and PH, elevated UA level was independently correlated with adverse outcomes, including all-cause mortality, cardiac death and HF hospitalization. Furthermore, NLR, a readily available systemic inflammatory biomarker, partially mediated the association between UA level and the risk of these adverse outcomes.

These findings highlight the importance of monitoring UA level and assessing systemic inflammation in patients with LVDD and PH. Comprehensive risk assessment, early detection and intervention to manage elevated UA level and reduce systemic inflammation may be crucial in improving patient outcomes and reducing the risk of adverse events. Further research is needed to confirm the results, explore the underlying mechanisms and evaluate the potential of targeted interventions in this patient population.

## Data Availability

The data that support the findings of this study are available on request from the corresponding authors.
